# Associating Genes and Protein Complexes with Disease via Network Propagation

**DOI:** 10.1371/journal.pcbi.1000641

**Published:** 2010-01-15

**Authors:** Oron Vanunu, Oded Magger, Eytan Ruppin, Tomer Shlomi, Roded Sharan

**Affiliations:** 1 School of Computer Science, Tel-Aviv University, Tel-Aviv, Israel; 2 Department of Computer Science, Technion, Haifa, Israel; University of British Columbia, Canada

## Abstract

A fundamental challenge in human health is the identification of disease-causing genes. Recently, several studies have tackled this challenge via a network-based approach, motivated by the observation that genes causing the same or similar diseases tend to lie close to one another in a network of protein-protein or functional interactions. However, most of these approaches use only local network information in the inference process and are restricted to inferring single gene associations. Here, we provide a global, network-based method for prioritizing disease genes and inferring protein complex associations, which we call PRINCE. The method is based on formulating constraints on the prioritization function that relate to its smoothness over the network and usage of prior information. We exploit this function to predict not only genes but also protein complex associations with a disease of interest. We test our method on gene-disease association data, evaluating both the prioritization achieved and the protein complexes inferred. We show that our method outperforms extant approaches in both tasks. Using data on 1,369 diseases from the OMIM knowledgebase, our method is able (in a cross validation setting) to rank the true causal gene first for 34% of the diseases, and infer 139 disease-related complexes that are highly coherent in terms of the function, expression and conservation of their member proteins. Importantly, we apply our method to study three multi-factorial diseases for which some causal genes have been found already: prostate cancer, alzheimer and type 2 diabetes mellitus. PRINCE's predictions for these diseases highly match the known literature, suggesting several novel causal genes and protein complexes for further investigation.

## Author Summary

Understanding the genetic background of diseases is crucial to medical research, with implications in diagnosis, treatment and drug development. As molecular approaches to this challenge are time consuming and costly, computational approaches offer an efficient alternative. Such approaches aim at prioritizing genes in a genomic interval of interest according to their predicted strength-of-association with a given disease. State-of-the-art prioritization problems are based on the observation that genes causing similar diseases tend to lie close to one another in a network of protein-protein interactions. Here we develop a novel prioritization approach that uses the network data in a global manner and can tie not only single genes but also whole protein machineries with a given disease. Our method, PRINCE, is shown to outperform previous methods in both the gene prioritization task and the protein complex task. Applying PRINCE to prostate cancer, alzheimer's disease and type 2 diabetes, we are able to infer new causal genes and related protein complexes with high confidence.

## Introduction

Associating genes with diseases is a fundamental challenge in human health with applications to understanding disease mechanisms, diagnosis and therapy. Linkage studies are often used to infer genomic intervals that are associated with a disease of interest. Prioritizing genes within these intervals is a formidable challenge and computational approaches are becoming the method of choice for such problems.

When one or more genes were already implicated in a given disease, the prioritization task is often handled by computing the functional similarity between a given gene and the known disease genes. Such a similarity can be based on sequence [1], functional annotation [2], protein-protein interactions [3],[4] and more (see [5] for a comprehensive review of these methods). When no causal genes are known, the prioritization is done by exploiting the modular view described above, comparing a candidate gene to other genes that were implicated in similar diseases.

Approaches in the latter category are often based on a measure of phenotypic similarity (see, e.g., [6],[7]) between the disease of interest and other diseases for which causal genes are known. This is motivated by the observation that genes causing the same or similar diseases often lie close to one another in a protein-protein interaction network [3],[5]. Lage et al. [7] score a candidate protein with respect to a disease of interest based on the involvement of its direct network neighbors in a similar disease. The protein and its high-confidence interactors are also suggested to form a putative protein complex that is related to the disease. Kohler et al. [8] group diseases into families. For a given disease, they employ a random walk from known genes in its family to prioritize candidate genes. Finally, Wu et al. [9] score a candidate gene 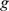 for a certain disease 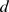 based on the correlation between the vector of similarities of 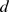 to diseases with known causal genes, and the vector of closeness in a protein interaction network of 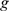 and those known disease genes. A recent follow-up work by Wu et al. introduces AlignPI, a method that exploits known gene-disease associations to align the phenotypic similarity network with the human PPI network [10]. The alignment is used to identify local dense regions of the PPI network and their associated disease clusters. The authors show the utility of their framework in causal gene prediction.

Most of these methods focus on prioritizing independent genes; however, in many cases, mutations at different loci could lead to the same disease. This genetic heterogeneity may reflect an underlying molecular mechanism in which the disease-causing genes form some kind of a functional module (e.g., a multi-protein complex or a signaling pathway) [7],[11]. For example, Fanconi anemia is a heterogeneous syndrome for which seven of its causing genes are known to form a protein complex which functions in DNA repair [12]. Thus, good prioritizations could potentially lead to the inference of larger disease-related machineries, revealing important mechanistic insights on the disease of interest.

While the above methods that integrate protein-protein interaction (PPI) information with a phenotypic similarity measure have been shown to outperform previous prioritization approaches, they are limited in their application. Specifically, both AlignPI and the method of Lage et al. consider only small localized regions of the PPI network and do not capture global network signals. The methods of Kohler et al. and Wu et al. tackle the prioritization task only, and do not suggest ways to reveal the protein modules that are affected in a given disease.

**Figure 1:**
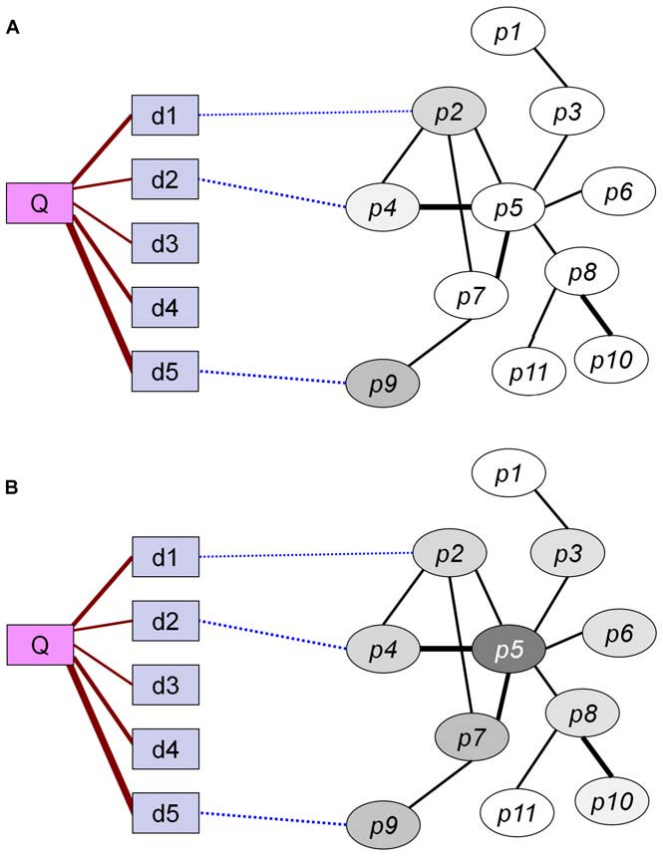
Illustration of the PRINCE algorithm. A query disease, denoted *Q*, has varying degrees of phenotypic similarity with other diseases, denoted *d1*–*d5* (marked with maroon lines, where thicker lines represent higher similarity). Known causal genes for these similar diseases are connected by dashed blue lines and used as the prior information. *p1*–*p11* comprise the protein set of a protein-protein interaction network, where interactions are marked with black lines and thicker lines denote edges with higher confidence. A scoring function that is smooth over the network is computed using an iterative network propagation method. At every iteration of the algorithm, each protein pumps flow to its neighbors and receives flow from them. Protein colors correspond to the flow they receive in a specific iteration, the darker the color the higher the flow. (A): the flow after the first iteration, representing the prior information. Only proteins *p2*, *p4* & *p9*, which are directly associated with similar diseases, have a positive incoming flow. (B): After several iterations, the amount of flow to each node converges, and the resulting flow, used to score the proteins, appears to be smooth over the network. *p5* emerges as the best causal gene candidate for disease *Q*, as it interacts with both *p2* and *p4*.

In this work we tackle both challenges. We present a novel network-based approach for predicting causal genes and protein complexes that are involved in a disease of interest. The method, which is called PRINCE (PRIoritizatioN and Complex Elucidation), generalizes the network-based approaches above by both considering the network signal in a global manner and going beyond single genes to the modules that are affected in a given disease. It receives as input a disease-disease similarity measure and a network of protein-protein interactions. It uses a propagation-based algorithm, a preliminary version of which appeared in [13], to infer a strength-of-association scoring function that is smooth over the network (i.e., adjacent nodes are assigned similar values) and exploits the prior information on causal genes for the same disease or similar ones. This process is illustrated in Figure 1. This scoring is then used in combination with a PPI network to infer protein complexes that are involved in the given disease.

We apply our method to analyze disease-gene association data from the Online Mendelian Inheritance in Man (OMIM) [14] knowledgebase. We test, in a cross-validation setting, the utility of our approach in prioritizing genes for all diseases with at least one known gene. We compare the performance of our method to two state-of-the-art, recently published methods [8],[9]. In all of our tests PRINCE outperforms the other methods by a significant margin. We then use our method to associate protein complexes with disease. The complexes that we recover are shown to be highly coherent in terms of the function, expression and conservation of their member proteins. According to these measures the collection of protein complexes we infer significantly outperforms a previous collection suggested by Lage et al. [7], in which each complex was limited to a protein and its direct interactors. Our complete set of predictions of gene- and protein-complex associations is available in the Supplementary Material (Suppl. Datasets S1, S2, S3).

We demonstrate the power of PRINCE by studying in detail three multi-factorial diseases for which some causal genes have been mapped already: Prostate Cancer, Alzheimer Disease and Non-insulin-dependant Diabetes Mellitus (Type 2). For each disease we investigate PRINCE's top-10 predictions when considering the entire network, and when limiting the search to genomic intervals that have been associated with the disease. 69% of these predictions are validated in the literature (using independent data), leaving 18 suggestions for novel causal genes.

## Results/Discussion

We designed a novel gene prioritization function, which integrates protein-protein interaction (PPI) information with a disease similarity metric to score the strength-of-association of proteins with a disease of interest. The scoring is designed to be smooth over the PPI network, meaning that adjacent nodes are assigned with similar values, and to exploit prior information on the involvement of proteins in the same or similar diseases. As further detailed in the Methods section, the scoring is done by simulating an iterative process where proteins for which prior information exist, pump flow to their network neighbors. In addition, every protein propagates the flow received in the previous iteration to its neighbors. The final score of each protein is determined by the amount of flow it has, which is guaranteed to converge.

### Comparison to other methods

In order to perform a comprehensive comparison of our approach to extant ones on the same input data, we reimplemented two state-of-the-art global approaches for gene prioritization introduced earlier: the random-walk based method of [8] and the Cipher algorithm [9]. We could not reimplement the method of Lage et al. [7], as it has many parameters that had to be returned to fit our data, and a code for this method was not readily available.

To evaluate the performance of the different methods, we used a leave-one-out cross validation procedure. In each cross-validation trial, we removed a single disease-protein association from the data, and each algorithm was evaluated by its success in reconstructing the hidden association, i.e., by the rank it assigned to a protein when querying the disease it is associated with (for further details on the cross-validation process see Methods). To simulate the case of prioritizing proteins encoded by genes inside a linkage interval, we followed [8] and artificially constructed for each protein associated with a disease an interval of size 100 around it. We evaluated the performance of an algorithm in terms of overall precision versus recall when varying the rank threshold 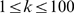. *Precision* is the fraction of true gene-disease associations that ranked within the top 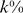 in the corresponding trial of the cross validation procedure. *Recall* is the fraction of trials in which the hidden association was recovered as one of the top 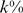 scoring ones.

**Figure 2:**
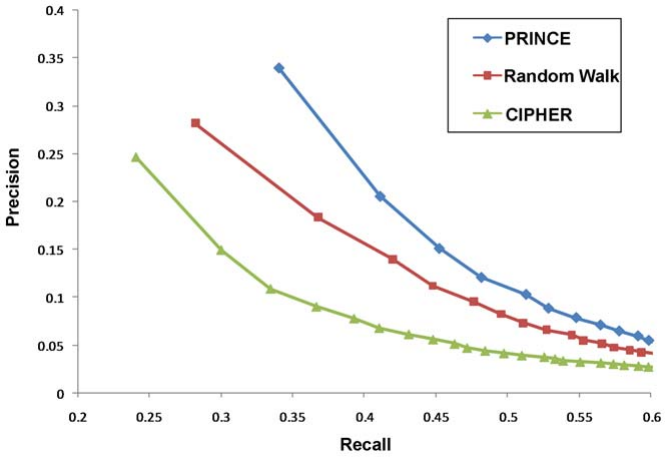
A comparison of prioritization algorithms. Performance comparison for PRINCE, Random Walk and CIPHER in a leave-one-out cross-validation test over 1,369 diseases with a known causal gene. The figure shows recall versus precision when considering the top 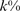 proteins for various values of 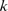.

We tested our method on all 1,369 diseases with a known causal gene in the OMIM database. The results of applying the different methods are depicted in Figure 2. Our algorithm achieved the best performance, ranking the correct gene as the top-scoring one in 34% of the cases. Random-walk and Cipher methods achieved inferior results with 28.8% and 24.7% success rates, respectively. This trend was maintained when performing 2-fold, 5-fold and 10-fold cross validation (Suppl. Figure S1).

Interestingly, even though our score does not directly indicate the probability of a successful prediction, we noticed a significant difference in the score distribution of top-1 correct predictions and top-1 incorrect prediction in the cross validation setting (see Suppl. Figure S2). Namely, about 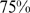 of our correct top-1 predictions received a score higher than 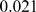, whereas about 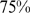 of our incorrect top-1 predictions received a score lower than that value. In the top-1 case, if all of the predictions with a score lower than 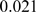 are discarded, PRINCE's precision is boosted to 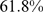, whereas the recall decreases to 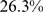.

To further validate the predicted associations, we collected recently published gene-disease associations that were not part of our original data set. We obtained 51 new associations for 47 diseases with previously known causal genes, and 10 new associations for diseases where the causal gene was unknown at the time of the original data collection. On the first association set, PRINCE ranked one of the newly associated genes as the top scoring one in 20 of the 47 diseases (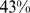). On this set, PRINCE significantly outperforms CIPHER and compares favorably to Random Walk (Suppl. Figure S3), providing higher precision and recall for 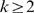. On the second association set, PRINCE ranked the newly associated gene as the top scoring one in two of the ten diseases, and ranked the true causal gene higher than or equal to the other methods in four additional cases, thus providing the best average ranking (Suppl. Table S1).

### Inferring novel causal genes for Prostate Cancer, Alzheimer and Diabetes

Having validated our method, we proceeded to execute our algorithm on specific multifactorial diseases that are linked to multiple genomic regions. We selected Prostate Cancer (MIM: 176807), Alzheimer's disease (MIM: 104300) and Diabetes Mellitus, type 2 (MIM: 125853) as our three case studies. We ranked candidate genes both over the entire PPI network, and over genomic intervals to which the phenotype has been mapped but no causal gene was identified, and analyzed our top-10 predictions in each case (Suppl. Table S2).

We checked whether our predicted genes were already found to be involved with their query disease by searching online databases [14]–[16] and scientific publications. In all of the three test cases, the vast majority of top candidate genes over the entire network were already known to be involved with the disease. These often included the ‘usual suspects’ for the relevant disease family. For example, the top predictions for Prostate Cancer included *BRCA1*, *TP53* and *NBN*, which are tumor suppressors involved in several types of cancer. Over half of the top candidates from the associated intervals were already implicated in the corresponding diseases. Our ranking provides further support for their involvement in the investigated diseases. In addition, PRINCE yields several top scoring candidates that were not previously associated with these diseases, providing viable candidates for further research.

Going beyond the above three test cases, we applied our algorithm to all 916 disorders in OMIM with an associated interval and for which no causal gene is known. The complete set of results is provided in the Supplementary Material (Suppl. Datasets S1).

### Associating protein complexes with disease

Often, as alluded to above, mutations in multiple proteins that form a protein complex or a pathway may lead to the same disease. Thus, we sought to exploit the prioritization function we have developed for the complex inference task. To this end, starting with the set of proteins whose prioritization score is above a threshold, we look for densely connected subsets that may form a protein complex. The search is aided by a likelihood-based scoring of protein complexes that takes into account the reliability of the PPI interactions and the degrees of the network proteins (Methods). As we show in Suppl. Figure S4 and S5, this score correlates well with the coherency of the identified complexes (see below). Applying this scheme to the OMIM diseases we predicted 566 complexes for diseases in which a causal gene is known and 137 complexes for diseases for which only an associated genomic interval is known.

To test the biological plausibility of the identified complexes we evaluated their coherency with respect to several attributes of their member proteins (Methods). These measures quantify the extent to which proteins in a complex share the same functional annotation, have similar expression patterns under multiple conditions, and have similar phylogenetic profiles, respectively. As a baseline, we compared these measures with those computed for: (i) a set of manually annotated protein complexes obtained from the Gene Ontology (GO) annotation [17]; (ii) a set of protein clusters that are not necessarily disease-related, obtained by applying the MCL algorithm [18] to the PPI network; and (iii) a set of predicted disease-related complexes (Lage et al. [7])(Methods).

To allow a fair comparison between our results and those of Lage et al., we focused on a subset of the identified complexes of the same size as that provided by Lage et al. (80 for the case of a known causal gene, and 59 for the case of a known locus; Methods). The subset was constructed by computing the likelihood score of each complex and choosing the highest ranking complexes.

We found that the complexes predicted using our propagation approach exhibited higher coherencies than the collection of Lage et al. with respect to most measures (with the exception of conservation coherency in the known-locus case). Notably, both our collection and that of Lage et al. outperformed the PPI-based collection produced by MCL, demonstrating the importance of the disease association data in the protein complex inference process. Moreover, our results were comparable to, and in some cases better than, the manually curated collection, again testifying to its high quality. These results are summarized in Table 1.

**Table 1:** Coherency comparison of different protein complex collections.

	Functional coherency (%)	Expression coherency (%)	Conservation coherency (%)
Known complexes	88.7	**47.4**	1.6
PPI-based complexes	48.1	12.4	0.2
Lage et al., gene known	77.5	18.9	3.75
Lage et al., locus known	74.6	18.2	6.8
PRINCE, gene known	**95**	43.8	**17.5**
PRINCE, locus known	89	35.6	1.7

Percentages represent the fraction of complexes whose coherency score passes a certain significance threshold (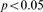 after correcting for multiple hypothesis testing). The best result in each column appears in bold.

As a further validation of the complexes inferred by PRINCE, we searched OMIM for evidence for the possible involvement of the proteins of a complex in the diseases associated with it. Specifically, for each complex, we scanned the OMIM entries of the diseases associated with at least one complex member. For each such disease, we checked whether any complex member that is not known to be associated with that disease, is mentioned in its entry. We found such support for 61% of the predicted complexes, with an average of 3.6 genes per complex whose involvement was corroborated in this manner. For comparison purpose, we permuted the gene names and repeated the analysis on the resulting random complexes. Only 7% of these random sets were supported by OMIM, with an average of 1.6 evidences per set.

**Figure 3:**
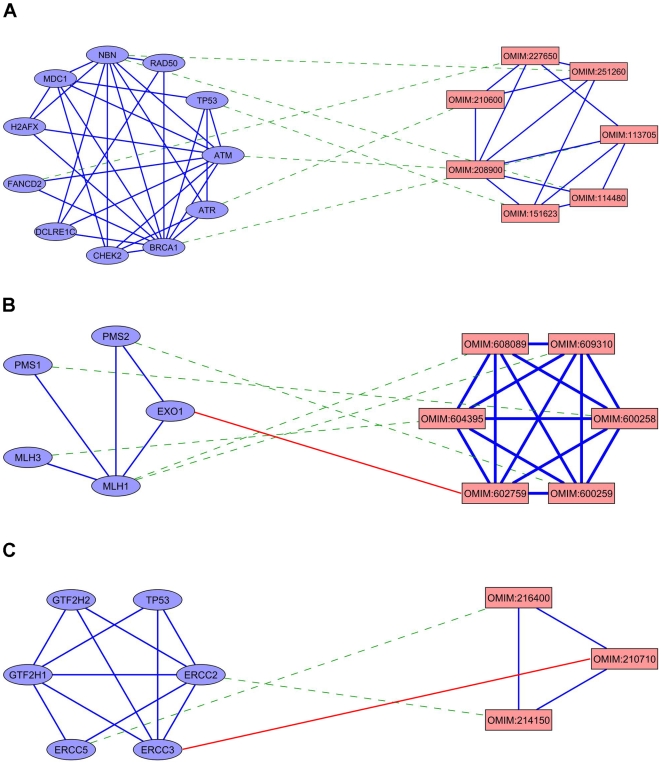
Case studies of inferred complexes. Examples of inferred protein complexes and their associated diseases. Circular nodes represent proteins and their connecting edges represent protein-protein interactions. Diseases are denoted by square nodes, connected by phenotypic similarity edges. Green dashed edges represent known gene-disease associations; red edges connect a disease to a gene that lies within its associated genomic interval. The complexes were generated for the query diseases (A) Ataxia-Telangiectasia, (B) Hereditary Prostate Cancer type 8 and (C) MOPD-I.

Three example putative protein complexes and their associated diseases are shown in Figure 3. The first putative protein complex (Figure 3(a)) was generated for the query disease Ataxia-Telangiectasia (MIM:208900), which is associated with the gene *ATM*. The putative complex contains 11 proteins which are all known to be involved in response to DNA damage stimulus. Except for *CHEK2*, all of them are directly involved in DNA repair. All 7 diseases associated to those genes (among them are Breast Cancer, Li-Fraumeni syndrome and Fanconi Anemia) are known to be tightly coupled with mutations in DNA-repair related genes. In this specific case it may be that these proteins do not form a single complex in-vivo, but rather span a dense region of the PPI network due to their central role as master regulators (especially ATM and TP53) of reactions to DNA damage.

The second complex (Figure 3(b)) was generated for the query disease Hereditary Prostate Cancer type 8 (HPC8, MIM:602759), for which the causal gene is presently unknown. The complex's proteins are associated with several Colorectal Cancer variants and Endometrial cancer. The genes associated with the Colorectal and Endometrial cancers are from the *MLH* (MutL analog) and *PMS* families which are involved in DNA mismatch repair. *MLH1* and *PMS2* form a Heterodimer, which interacts via *MLH1* with *EXO1* (Exonuclease1), which also participates in DNA mismatch repair. The gene coding for *EXO1* is located at genetic locus 1q43, which lies within the region associated with HPC8 (1q42.2–q43). Moreover, *EXO1* was ranked first by PRINCE in this interval. In this case, the inferred protein complex provides support also to the prediction that *EXO1* is a causal gene for prostate cancer (MIM: 176807) discussed in the previous subsection.

The last complex (Figure 3(c)) was generated for the query disease Microcephalic Osteodysplatic Primordial Dwarfism (MOPD-I, MIM:210710), which has no known causal genes. Two of the predicted complex's genes are associated with two hereditary diseases characterized by developmental delay and physical deformations: *ERCC5* with Cockayne Syndrome type A, and *ERCC2* with Cerebrooculofacioskeletal Syndrome 1. The genes in the complex are all involved in DNA damage repair: *ERCC2*, *ERCC3*, *GTF2H1* and *GTF2H2* are subunits of the core-TFIIH basal Transcription Factor, and *ERCC5* forms a stable complex with TFIIH enabling recruitment of the Transcription Factor for repairing UV damage [19]. *ERCC3*, one of the predicted complex's members, lies within the genetic locus associated with MOPD-I, and is ranked as the best causal gene candidate for MOPD-I among the genes at that locus by PRINCE.

### Conclusions

PRINCE is a powerful method for prioritizing genes and protein complexes for a disease of interest. We have demonstrated its power both in a cross validation setting and by closely examining its predictions over complex, polygenic hereditary diseases. Key to its successful application is its global network approach, combined with a novel normalization of protein-protein interaction weights and disease-disease similarities.

While the results of PRINCE are promising, several of its limitations should be acknowledged. First, PRINCE relies on prior phenotypic information, which limits its application to diseases that are phenotypically similar to diseases with known causal genes. Second, PRINCE uses known gene-disease associations in its computation, but other relevant data, such as genes that are differentially expressed in the disease state, are not taken into account. Combining such data into the prioritization process, e.g., using the method of [20], could increase the prediction power. Last, PRINCE depends on accurate and comprehensive protein-protein interaction data. As such data accumulate, the applicability and accuracy of PRINCE are expected to grow.

## Methods

### Computing the prioritization function

The input to a gene prioritization problem consists of a set 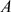 of gene-disease associations; a query disease 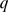; and a protein-protein interaction network 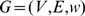, where 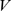 is the set of proteins, 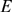 is the set of interactions and 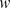 is a weight function denoting the reliability of each interaction. The goal is to prioritize all the proteins in 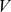 (that are not known to be associated with 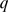) with respect to 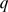.

For a node 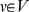, denote its direct neighborhood in 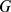 by 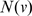. Let 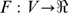 represent a prioritization function, i.e., 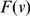 reflects the relevance of 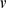 to 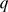. Let 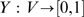 represent a prior knowledge function, which assigns positive values to proteins that are known to be related to 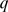, and zero otherwise.

Intuitively, we wish to compute a function 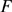 that is both smooth over the network, i.e., adjacent nodes are assigned with similar values, and also respects the prior knowledge, i.e., nodes for which prior information exists should have similar values of 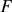 and 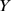. Formally, we express the requirements on 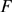 as a combination of these two conditions:
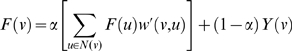
(1)where 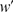 is a normalized form of 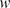 (described below). The parameter 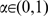 weighs the relative importance of these constraints with respect to one another.

The requirements on 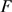 can be expressed in linear form as follows:
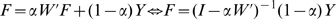
(2)where 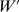 is a 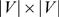 matrix whose values are given by 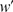, and 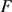 and 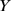 are viewed here as vectors of size 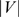. We require the eigenvalues of 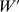 to be in 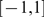. Since 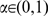, the eigenvalues of 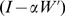 are positive and, hence, 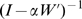 exists.

While the above linear system can be solved exactly, for large networks an iterative propagation-based algorithm works faster and is guaranteed to converge to the system's solution. Specifically, we use the algorithm of Zhou et al. [21] which at iteration 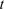 computes
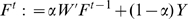
where 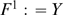. This iterative algorithm can be best understood as simulating a process where nodes for which prior information exists pump information to their neighbors. In addition, every node propagates the information received in the previous iteration to its neighbors.

We chose to normalize the weight of an edge by the degrees of its end-points, since the latter relate to the probability of observing an edge between the same end-points in a random network with the same node degrees. Formally, define a diagonal matrix 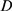 such that 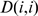 is the sum of row 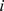 of 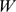. We set 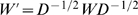 which yields a symmetric matrix where 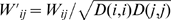. Note that 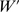 is similar to the stochastic matrix 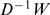. Since similar matrices have the same eigenvalues, and since a stochastic matrix's eigenvalues are in 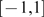 (according to the Perron-Frobenius theorem), the eigenvalues of 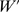 are indeed in 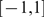.

### Incorporating disease similarity information

To determine the prior information vector 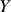, we used the similarity metric computed by van Driel et al. [6]. This metric spans 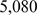 diseases in the OMIM [14] knowledgebase and is based on their medical subject headings description.

van Driel et al. tested the predictive power of different ranges of similarity values by calculating the correlation between the similarity of two diseases and the functional relatedness of their causal genes. According to their analysis, similarity values in the range 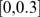 are not informative, while for similarities in the range 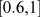 the associated genes show significant functional similarity.

These empirical findings motivated us to represent our confidence that two diseases are related using a logistic function 
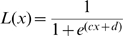
, such that for 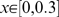, 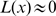, and for 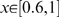, 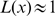. This implies that 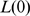 needs to be close to 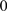. We set 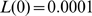, which determines 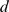 as 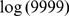, and tuned the parameter 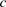 using cross validation (see Parameter Tuning Section below).

We used 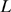 to compute the prior knowledge 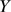 in the following way: for a query disease 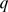 and a protein 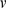 associated with a disease 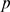, we set 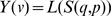, where 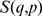 is the similarity between 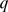 and 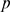. If 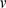 is associated with several diseases, we choose the disease 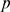 which is the most similar to 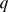.

### Experimental setup

We extracted 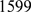 known disease-protein associations from GeneCards [15] spanning 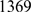 diseases and 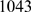 proteins. We considered only disease-protein relations that included proteins from the network and such that the relations are known to be causative to avoid associations made by circumstantial evidence.

We constructed a human PPI network with 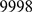 proteins and 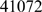 interactions that were assembled from three large scale experiments [22]–[24] and the Human Protein Reference Database (HPRD) [25]. The interactions were assigned confidence scores based on the experimental evidence available for each interaction using a logistic regression model adapted from [26]. We used the obtained scores to construct the adjacency matrix 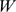.

To simulate the case of prioritizing proteins encoded by genes inside a linkage interval, we followed [8] and artificially constructed for each protein associated with a disease an interval of size 100 around it. We used the protein scores obtained from the output of the algorithm to prioritize proteins residing in that interval.

To evaluate the performance of the different methods in predicting gene-disease association, we used a leave-one-out cross validation procedure. In each cross-validation trial, we removed a single disease-protein association 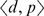 from the data, and in addition all other disease-protein associations involving protein 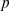. An algorithm was evaluated by its success in reconstructing the hidden association, i.e. by the rank it assigned to protein 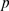 when querying disease 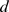. The reason we hid all associations of 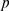 was to avoid “easy” cases in which 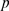 is also associated with other diseases that are very similar to 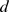.

### Parameter tuning for gene prioritization

Our algorithm has three parameters that should be tuned: (i) 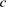 – the parameter controlling the logistic regression transformation; (ii) 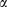 – controlling the relative importance of prior information in the association assignment; and (iii) the number of propagation iterations employed. We tested the effect of these parameters on the performance of the algorithm in a cross validation setting. The precision-recall plots for the general disease case are depicted in Suppl. Figure S6. By this figure, the optimal regression coefficient is 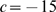, implying that similarity values below 0.3 are assigned with very low probability (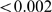), in accordance with the analysis of [6]. The algorithm is not sensitive to the actual choice of 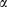 as long as it is above 0.5 (panel b). Finally, the algorithm shows fast convergence, achieving optimal results after ten iterations only (panel c).

### Implementation of random-walk and Cipher

The random-walk based approach requires disease grouping information. To allow it to run on the more comprehensive disease similarity data we had, we generalized the approach to use these similarities (transformed by the logistic function 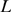) as initial probabilities for the random walk. The parameter 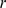 of the method, which controls the probability for a restart, as well as our transformation parameter 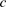, were optimized using cross-validation (as in the Parameter Tuning Section above). Note that Kohler et al. suggested a second, diffusion-kernel based approach, which was shown to be inferior to the random walk one, hence we did not include it in our comparison. Also note that our propagation-based method reduces to a random walk under appropriate transformations of the edge weights and prior information.

The Cipher method [9] is based on computing protein closeness in a PPI network. Two variants of the algorithm were suggested: Cipher-DN, which considers only direct neighbors in the closeness computation, and Cipher-SP, which is based on a shortest path computation. The former was shown to outperform the latter, and hence we implemented this variant (Cipher-DN) only.

### Identifying protein complexes

Given a disease and a prioritization score for all the network proteins, we aim at inferring densely connected protein complexes that contain high scoring proteins. To this end, we start with the top 100 scoring proteins within the entire network as complex seeds (The method is not sensitive to the number of initial top scoring proteins, and produces similar results for numbers in the range 50–150; data not shown). We filter all seeds whose score is below a prespecified threshold 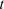 or that were already associated with the disease in a previously detected complex. To each seed we iteratively add a neighboring protein with the highest score, as long as this score is greater than 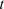, and up to 20 proteins per seed (about twice the average size of known protein complexes; a similar bound was used in previous works [26],[27]). At this stage, in the case that the query disease has no known gene, but has an interval associated to it, the computed complex is discarded if it contains no member from that interval.

After an initial list of putative complexes is formed, a refinement phase takes place where proteins are removed from a putative complex to ensure that not only is the suggested complex disease-related but also its member proteins are densely interacting and, thus, constitute a good candidate for a complex. To this end, we use the following likelihood-based scoring scheme taken from [28]:
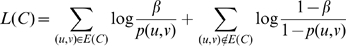
where 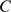 is a putative complex and 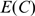 are its edges. Briefly, the score is the log likelihood ratio between a protein complex model (assuming that every two proteins in a complex should interact with a high probability 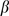, independently of all other pairs) and a random set model (where connections in the sub-network arise at random, with a probability proportional to the proteins' degrees). This score was further enhanced, as in [28] to accommodate for information on the reliability of interactions. In brief, the interaction status of every protein pair was treated as a noisy observation, and its reliability was combined into the likelihood score. The 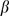 parameter of the scoring scheme was set to 0.9, although results were not sensitive to the actual parameter used as shown in Suppl. Table S3.

At each refinement step, we search for a protein whose removal increases the score the most while maintaining the connectivity of the candidate complex. The refinement is done until no score increase is possible (while maintaining connectivity). We filter candidate complexes with less than four proteins (to ensure statistical significance) or with 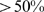 overlap with higher-scoring candidates.

For identifying complexes we use the same 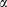 and 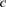 values we used for prioritization, which were tuned using cross-validation. An additional parameter, 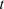, is used as a threshold that defines the minimal score (computed using propagation) needed for a protein to be included in any identified complex. This parameter was tuned separately for the case in which a causal gene for the query disease is known and for the case that no causal gene is unknown. The tuning aimed to obtain a collection of complexes whose average size is similar to that of the manually curated GO complexes (8.85 after filtering complexes with 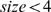 or 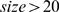). The resulting value of 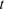 is 0.1 (average size of 8.3) for the case where a causal gene is known, and 0.015 (average size of 8.5) for the case where no causal gene is known.

### Evaluation of protein complex predictions

We compared the protein complexes inferred by PRINCE to three other collections:

- A gold standard set of 70 manually annotated protein complexes retrieved from GO [17], by considering the gene product associations of all terms that descended from the ‘Protein Complex’ term (GO:0043234).
- A collection of 160 clusters of proteins, obtained by applying Markov Clustering [18] to the PPI network and sampling 160 of its clusters while maintaining the same size distribution as the 80 protein complexes inferred by PRINCE for the known causal gene case.
- The collection of complexes published by Lage et al [7]. We filtered this collection by removing complexes of 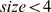 or 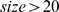. We further filtered overlapping complexes as described above. The filtering resulted in 80 complexes for the known causal gene case, and 59 for diseases for which no causal gene is known.

Following [29], we evaluated the different collections using three coherency measures:

#### Functional coherency

The percent of functionally coherent complexes based on the GO process annotation. For a given complex 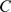 and a given term 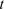, let 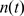 be the number of proteins in 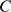 that are annotated with 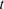 (or with a more specific term). Let 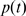 be the hypergeometric probability for observing 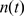 or more proteins annotated with term 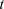 in a protein subset of size 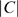. Having found a term 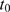 with minimal probability 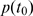, the score was set to the empirical p-value of the enrichment under term 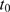, computed by comparing 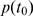 with the analogous probabilities for 10,000 random protein sets of size 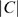. These latter p-values were corrected for multiple complex testing using the False Discovery Rate (FDR) procedure [30].

#### Expression coherency

The percent of expression coherent complexes. Each protein complexes was scored by the mean pairwise Pearson correlation of gene expression profiles [31] among all its members. The statistical significance of the expression coherency score was computed by comparing it to similar scores obtained for randomly drawn protein sets of the same size. These p-values were further FDR-corrected for multiple complex testing.

#### Conservation coherency

The percent of conservation coherent complexes. Phylogenetic profiles (i.e., presence/absence patterns) of human genes in a set of 18 eukaryotic genomes were obtained from NCBI's HomoloGene database [32]. The conservation coherency of a cluster was defined as the mean pairwise Jaccard similarity among the phylogenetic profiles of the cluster's members. These scores were compared to those obtained for randomly drawn protein sets of the same size and FDR-corrected for multiple complex testing.

In all three cases, a complex was considered to be significantly coherent if its corrected p-value was below a threshold of 0.05.

### Hardware, performance and availability

The computational experiments were executed on a single core of an AMD Opteron(tm) 2382 processor 2.6 Ghz. The average runtime for completing the cross validation iterations or inferring protein complexes was 1–2 minutes. The code and data sets described herein are available upon request.

## Supporting Information

Figure S1 K-fold cross validation comparison of PRINCE, Random Walk and CIPHER. (a) 2-fold (b) 5-fold (c) 10-fold.
(0.14 MB JPG)

Figure S2 Log-score distribution for genes (a) correctly and (b) in-correctly ranked 1st by PRINCE during Leave-One-Out cross validation trials.
(0.01 MB PNG)

Figure S3 Performance comparison on 47 diseases with a known causal gene, for which another causal gene was recently discovered.
(0.02 MB PNG)

Figure S4 Biological plausability scores for complexes inferred from diseases with a known causal gene.
(0.06 MB JPG)

Figure S5 Biological plausability scores for complexes inferred from diseases with an associated genomic region and an unknown causal gene.
(0.06 MB JPG)

Figure S6 PRINCE algorithm parameters fine-tuning summary: (a) Performance comparison for various logistic regression parameters; (b) Performance comparison with varying iterations count; (c) Performance comparison for various values of alpha.
(0.16 MB JPG)

Table S1 Comparison of the ranking given by PRINCE, Random Walk and CIPHER to recently discovered causal genes for ten diseases for which no causal gene was known at the inception of this research.
(0.02 MB PDF)

Table S2 PRINCE's causal gene predictions for Prostate Cancer, Alzheimer's Disease and type 2 Diabetes.
(0.04 MB XLS)

Table S3 Comparing the effect of different values of beta on the inferred complexes, in terms of functional enrichment, expression coherency and conservation coherency.
(0.02 MB PDF)

Dataset S1 Known loci disorders. PRINCE's causal gene predictions for 916 OMIM disorders with an associated interval and without a known causal gene.
(0.94 MB TXT)

Dataset S2 Known gene complexes. 80 Protein Complexes inferred for disorders with a known causal gene.
(3 KB TXT)

Dataset S3 Known loci complexes. 59 Protein Complexes inferred for disorders with an unknown causal gene and an associated genomic interval.
(2 KB TXT)

## References

[1] George RA, Liu JY, Feng LL, Bryson RJ, Fetkin D (2006). Analysis of protein sequence and interaction data for candidate disease gene prediction.. Nucleic Acids Res.

[2] Perez-Iratxeta C, Bork P, Andrade-Navarro MA (2007). Update of the g2d tool for prioritization of gene candidates to inherited diseases.. Nucleic Acids Res.

[3] Oti M, Snel B, Huynen MA, Brunner HG (2006). Predicting disease genes using protein-protein interactions.. J Med Genet.

[4] Franke L, Bakel H, Fokkens L, de Jong ED, Egmont-Petersen M (2006). Reconstruction of a functional human gene network, with an application for prioritizing positional candidate genes.. Am J Hum Genet.

[5] Oti M, Brunner HG (2007). The modular nature of genetic diseases.. Clinical Genetics.

[6] van Driel MA, Bruggeman J, Vriend G, Brunner HG, Leunissen JAM (2006). A text-mining analysis of the human phenome.. Eur J Hum Genet.

[7] Lage K, Karlberg EO, Storling ZM, Olason PI, Pedersen AG (2007). A human phenome-interactome network of protein complexes implicated in genetic disorders.. Nat Biotech.

[8] Kohler S, Bauer S, Horn D, Robinson PN (2008). Walking the interactome for prioritization of candidate disease genes.. American journal of human genetics.

[9] Wu X, Jiang R, Zhang MQ, Li S (2008). Network-based global inference of human disease genes.. Mol Syst Biol.

[10] Wu X, Liu Q, Jiang R (2009). Align human interactome with phenome to identify causative genes and networks underlying disease families.. Bioinformatics.

[11] Brunner HG, van Driel MA (2004). From syndrome families to functional genomics.. Nat Rev Genet.

[12] D'Andrea AD (2003). The fanconi anemia/brca signaling pathway: disruption in cisplatin-sensitive ovarian cancers.. Cell cycle (Georgetown, Tex).

[13] Vanunu O, Sharan R (2008). A propagation based algorithm for inferring gene-disease associations.. Proceedings of the German Conference on Bioinformatics.

[14] Hamosh A, Scott AF, Amberger JS, Bocchini CA, McKusick VA (2002). Online mendelian inheritance in man (omim), a knowledgebase of human genes and genetic disorders.. Nucl Acids Res.

[15] Rebhan M, Chalifa-Caspi V, Prilusky J, Lancet D (1997). Genecards: integrating information about genes, proteins and diseases.. Trends in Genetics.

[16] Becker KG, Barnes KC, Bright T, Wang A (2004). The genetic association database.. Nature Genetics.

[17] The-Gene-Ontology-Consortium (2000). Gene ontology: tool for the unification of biology.. Nature Genetics.

[18] Enright AJ, Van-Dongen S, Ouzounis CA (2002). An efficient algorithm for large-scale detection of protein families.. Nucleic Acids Research.

[19] Thorel F, Constantinou A, Dunand-Sauthier I, Nouspikel T, Lalle P (2004). Definition of a short region of xpg necessary for tfiih interaction and stable recruitment to sites of uv damage.. Mol Cell Biol.

[20] Karni S, Soreq H, Sharan R (2009). A network-based method for predicting disease-causing genes.. Journal of Computational Biology.

[21] Zhou D, Bousquet O, Lal TN, Weston J, Scholkopf B (2003). Learning with local and global consistency.. http://citeseer.ist.psu.edu/zhou03learning.html.

[22] Rual JF, Venkatesan K, Hao T, Hirozane-Kishikawa T, Dricot A (2005). Towards a proteome-scale map of the human protein-protein interaction network.. Nature.

[23] Stelzl U, Worm U, Lalowski M, Haenig C, Brembeck FH (2005). A human protein-protein interaction network: a resource for annotating the proteome.. Cell.

[24] Ewing R, Chu P, Elisma F, Li H, Taylor P (2007). Large-scale mapping of human protein-protein interactions by mass spectrometry.. Mol Syst Biol.

[25] Peri S, Navarro JD, Kristiansen TZ, Amanchy R, Surendranath V (2004). Human protein reference database as a discovery resource for proteomics.. Nucleic Acids Res.

[26] Sharan R, Suthram S, Kelley RM, Kuhn T, McCuine S (2005). Conserved patterns of protein interaction in multiple species.. Proc Natl Acad Sci.

[27] Sharan R, Ideker T, Kelley B, Shamir R, Karp R (2005). Identification of protein complexes by comparative analysis of yeast and bacterial protein interaction data.. Journal of Computational Biology.

[28] Sharan R, Ideker T (2006). Modeling cellular machinery through biological network comparison.. Nat Biotech.

[29] Tan K, Shlomi T, Feizi H, Ideker T, Sharan R (2007). Transcriptional regulation of protein complexes within and across species.. Proc Natl Acad Sci.

[30] Benjamini Y, Hochberg Y (1995). Controlling the false discovery rate - a practical and powerful approach to multiple testing.. J Roy Stat Soc B Met.

[31] Levine D, Haynor DR, Castle JC, Stepaniants SB, Pellegrini M (2006). Pathway and gene-set activation measurement from mrna expression data: the tissue distribution of human pathways.. Genome Biology.

[32] Wheeler DL, Barrett T, Benson DA, Bryant SH, Canese K (2006). Database resources of the national center for biotechnology information.. Nucleic Acids Res.

