# Supplementary figures and images for: Associating Genes and Protein Complexes with Disease via Network Propagation
Source: PLoS Comput Biol. 2010 Jan 15;6(1):e1000641. doi: 10.1371/journal.pcbi.1000641 (PMC2797085; doi:10.1371/journal.pcbi.1000641)

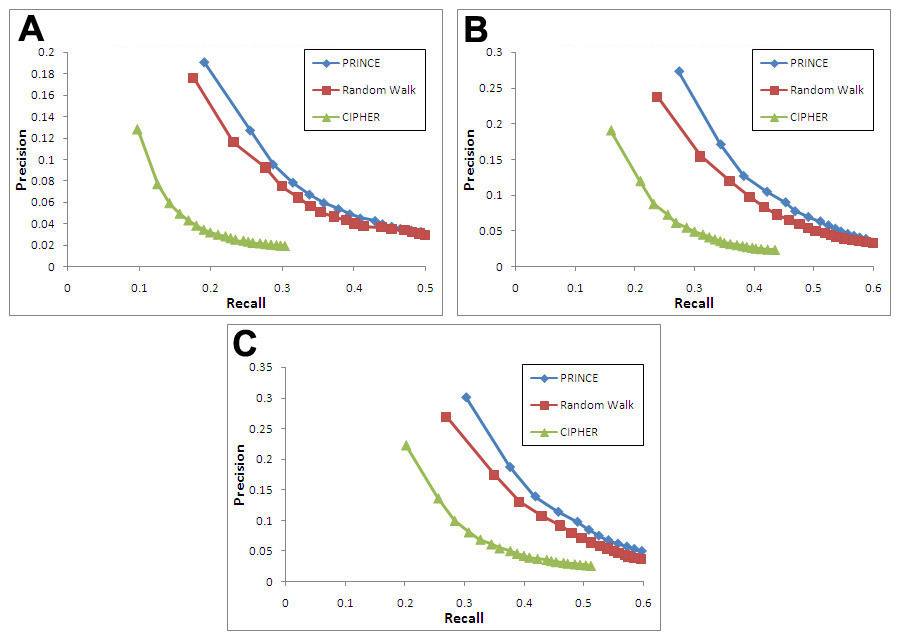

Supplement: Figure S1 — K-fold cross validation comparison of PRINCE, Random Walk and CIPHER. (a) 2-fold (b) 5-fold (c) 10-fold. (0.14 MB JPG) [file pcbi.1000641.s001.jpg]

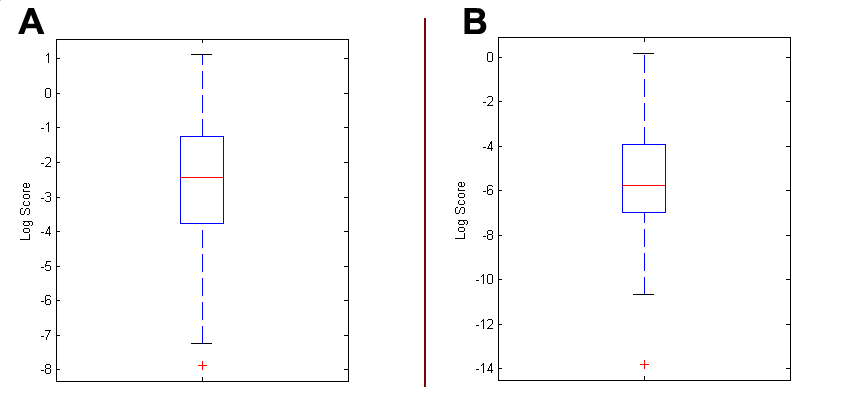

Supplement: Figure S2 — Log-score distribution for genes (a) correctly and (b) in-correctly ranked 1st by PRINCE during Leave-One-Out cross validation trials. (0.01 MB PNG) [file pcbi.1000641.s002.png]

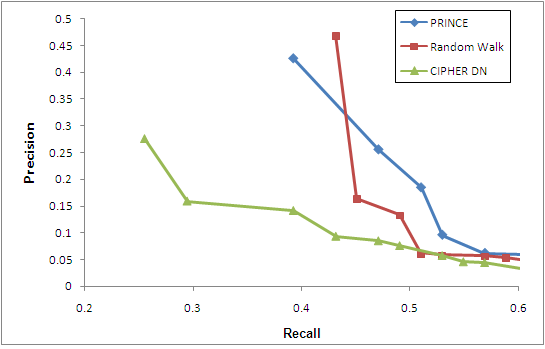

Supplement: Figure S3 — Performance comparison on 47 diseases with a known causal gene, for which another causal gene was recently discovered. (0.02 MB PNG) [file pcbi.1000641.s003.png]

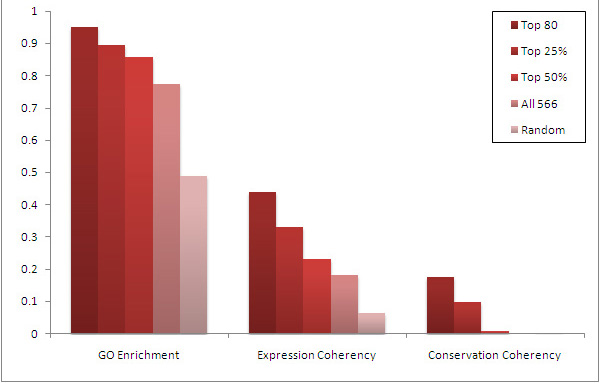

Supplement: Figure S4 — Biological plausability scores for complexes inferred from diseases with a known causal gene. (0.06 MB JPG) [file pcbi.1000641.s004.jpg]

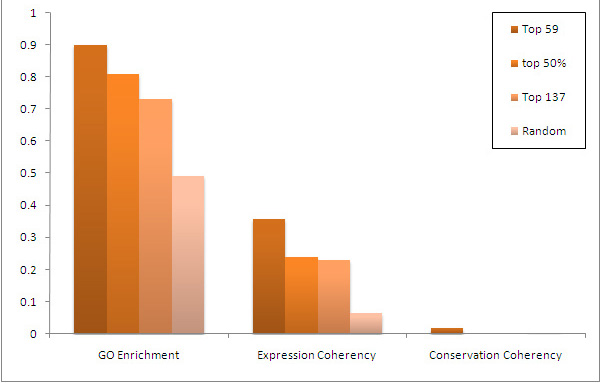

Supplement: Figure S5 — Biological plausability scores for complexes inferred from diseases with an associated genomic region and an unknown causal gene. (0.06 MB JPG) [file pcbi.1000641.s005.jpg]

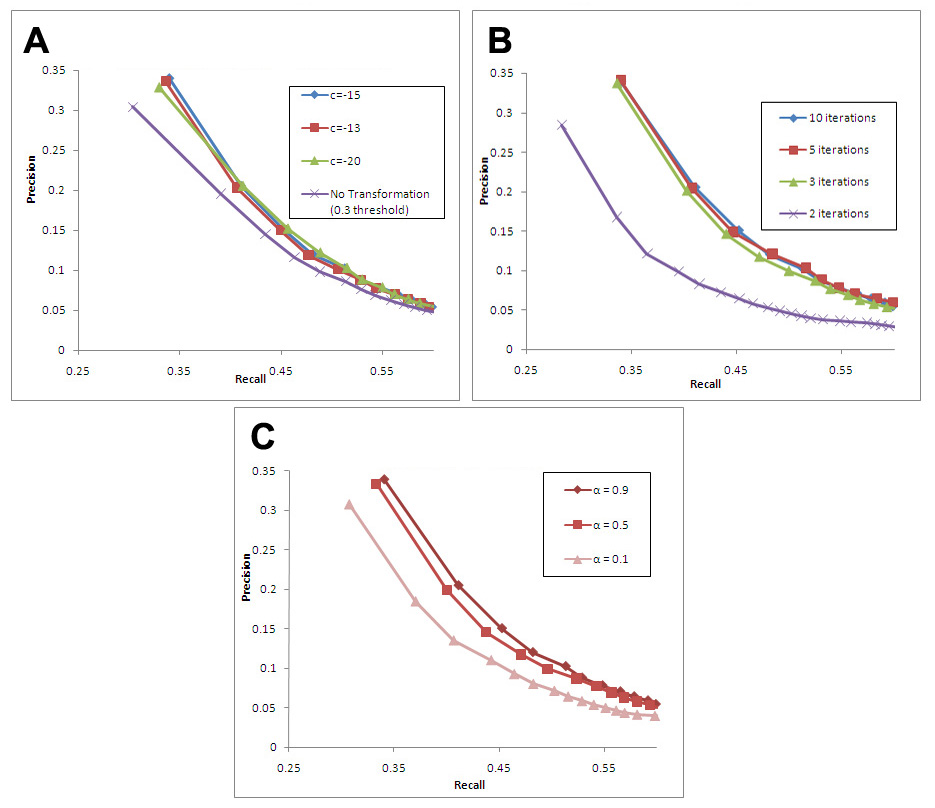

Supplement: Figure S6 — PRINCE algorithm parameters fine-tuning summary: (a) Performance comparison for various logistic regression parameters; (b) Performance comparison with varying iterations count; (c) Performance comparison for various values of alpha. (0.16 MB JPG) [file pcbi.1000641.s006.jpg]
