# Supplementary material for: Associating Genes and Protein Complexes with Disease via Network Propagation
Source: PLoS Comput Biol. 2010 Jan 15;6(1):e1000641. doi: 10.1371/journal.pcbi.1000641 (PMC2797085; doi:10.1371/journal.pcbi.1000641)
Supplement: Table S1 — Comparison of the ranking given by PRINCE, Random Walk and CIPHER to recently discovered causal genes for ten diseases for which no causal gene was known at the inception of this research. (0.02 MB PDF) [file pcbi.1000641.s007.pdf]

Disease (OMIM)	Causal gene (Entrez)	PRINCE	Random Walk	CIPHER
300600	CACNA1F (778)	**1**	**1**	5
150230	EXT1 (2131)	**1**	**1**	**1**
300048	FLNA (2316)	12	3	**1**
311070	PRPS (5631)	13	**7**	24
301835	PRPS (5631)	12	**8**	20
609923	TOPORS (10210)	35	39	**33**
601493	LDB3 (11155)	**8**	9	67
610448	TREX1 (11277)	**8**	10	**8**
610448	TREX1 (11277)	**3**	5	17
604432	TTBK2 (146057)	**69**	98	74

Table S1. Comparison of the ranking given by PRINCE, Random Walk and CIPHER to recently discovered causal genes for ten diseases for which no causal gene was known at the inception of this research. The best ranking for each disease is emphasized.
