# Supplementary material for: Associating Genes and Protein Complexes with Disease via Network Propagation
Source: PLoS Comput Biol. 2010 Jan 15;6(1):e1000641. doi: 10.1371/journal.pcbi.1000641 (PMC2797085; doi:10.1371/journal.pcbi.1000641)
Supplement: Table S3 — Comparing the effect of different values of beta on the inferred complexes, in terms of functional enrichment, expression coherency and conservation coherency. (0.02 MB PDF) [file pcbi.1000641.s009.pdf]

Beta	#Predicted complexes	GO enrichment (%)	Expression coherency (%)	Conservation coherency (%)
0.8	549	93.75	40	10
0.85	558	**95**	**45**	12.5
0.9	566	**95**	43.75	**17.5**
0.95	565	93.75	41.25	15

Table S3. Comparing the effect of different values of beta on the inferred complexes, in terms of functional enrichment, conservation coherency and expression coherency.
